# New SHapley Additive ExPlanations (SHAP) Approach to Evaluate the Raw Materials Interactions of Steel-Fiber-Reinforced Concrete

**DOI:** 10.3390/ma15186261

**Published:** 2022-09-09

**Authors:** Madiha Anjum, Kaffayatullah Khan, Waqas Ahmad, Ayaz Ahmad, Muhammad Nasir Amin, Afnan Nafees

**Affiliations:** 1 Department of Computer Engineering, College of Computer Science and Information, Technology, King Faisal University, Al-Ahsa 31982, Saudi Arabia; 2 Department of Civil and Environmental Engineering, College of Engineering, King Faisal University, Al-Ahsa 31982, Saudi Arabia; 3 Department of Civil Engineering, COMSATS University Islamabad, Abbottabad 22060, Pakistan; 4 MaREI Centre, Ryan Institute and School of Engineering, College of Science and Engineering, National University of Ireland Galway, H91 TK33 Galway, Ireland

**Keywords:** steel fiber, building material, flexural strength, fibers, concrete, mortar, hybrid

## Abstract

Recently, artificial intelligence (AI) approaches have gained the attention of researchers in the civil engineering field for estimating the mechanical characteristics of concrete to save the effort, time, and cost of researchers. Consequently, the current research focuses on assessing steel-fiber-reinforced concrete (SFRC) in terms of flexural strength (FS) prediction by employing delicate AI techniques as well as to predict the raw material interaction that is still a research gap. In this study, the FS of SFRC is estimated by deploying supervised machine learning (ML) techniques, such as DT-Gradient Boosting, DT-XG Boost, DT-AdaBoost, and DT-Bagging. In addition to that, the performance model is also evaluated by using R^2^, root mean square error (RMSE), and mean absolute error (MAE). Furthermore, the k-fold cross-validation method is also applied to validate the model’s performance. It is observed that DT-Bagging with an R^2^ value of 0.95 is superior to DT-XG Boost, DT-Gradient Boosting, and DT-AdaBoost. Lesser error MAE and RMSE and higher R^2^ values for the DT-Bagging model show the enhanced performance of the model compared to the other ensembled approaches. Considerable conservation of time, effort, and cost can be made by applying ML techniques to predict concrete properties. The evaluation of the outcome depicts that the estimated results of DT-Bagging are closer to the experimental results, indicating the accurate estimation of SFRC flexural strength. It is further revealed from the SHapley Additive exPlanations (SHAP) study that the volumetric content of steel fiber highly and positively influences the FS of SFRC.

## 1. Introduction

Modern artificial intelligence (AI) approaches are effective for evaluating the difficult problems of the engineering domain. By using these techniques, the output end products can be predicted with a set of input factors. Single-model-based standalone and ensemble, i.e., AdaBoost and bagging, methods are the two primary methods of machine learning (ML) that are used for predicting the properties of concrete. As per the available literature, the prediction performance of ensemble methods is better than the individual machine learning algorithm. Chaabene et al. [1] predicted the mechanical characteristics of concrete by employing ML approaches. Likewise, abundant literature is available on the utilization of ML for predicting different concrete types, such as recycled aggregates [2,3,4,5], self-healing [6], materials-integrated [7], and high-performance [8,9,10,11,12] concretes. In a study conducted by Han et al. [9] for predicting high-performance concrete strength via ML approaches, the considered input parameters were i. cement, ii. fine and coarse aggregates, iii. water, iv. fly ash, v. ground-granulated blast furnace slag, and vi. The ageing period. The study concluded with the highly precise prediction of high-performance concrete strength using the developed ML model. The toughness, ductility, resistance to cracks, mechanical properties, and fatigue resistance of concrete can be enhanced by adding fibers [13,14,15,16,17,18,19,20,21,22,23,24,25]. Incorporating steel fibers in cementitious composites can enhance their post-cracking behavior and toughness [26,27,28,29]. Different fiber types, such as steel, and artificial and natural fibers, have been explored in various studies for their potential application as construction materials [25,30,31,32,33,34,35]. In SFRC, additional estimation factors regarding regular concrete are considered, such as the aspect ratio of steel fibers, their type, and the volumetric percentage content. However, the development of appropriate estimation models for SFRC is relatively new. Accordingly, conventional regression models (linear and nonlinear) are employed to determine SFRC flexural strength (FS).

The properties of different concrete types can now be precisely predicted by applying ML approaches. Significant effort, time, and cost are needed during experimental investigations. Hence, to save time it is necessary to develop data-modelling-based algorithms to identify closely linked independent parameters. The necessity is to employ AI techniques to estimate the properties of novel concrete types. ML techniques to predict SFRC FS are an effective alternative to save the cost, time, and effort required for the experimental setup. Accordingly, in the current work, the FS of steel-fiber-reinforced concrete (SFRC) is predicted by using artificial intelligence-based machine learning methods. Subsequently, in this work, the employment of ensemble ML models, such as gradient boosting, AdaBoost, XG Boosting, and bagging ensembled ML approaches, is done to achieve the study objectives. Moreover, the application of statistical checks is also done for the testing of models in addition to the comparison of all the applied models [36,37,38,39]. A model with the best performance is proposed based on performance due to applied statistical checks for the prediction of SFRC properties. Afterwards, a game theory approach [40], named SHapley Additive exPlanations (SHAP), is also employed to obtain an enhanced description of applied ML models by global features influences classification and interactions/dependencies. A novel knowledge era is identified by this method in terms of SFRC ingredients’ influences on FS. It would assist the researcher’s ability to identify adequate SFRC mix combinations and quickly estimate its FS without even performing experimental procedures for trials. It would also aid the upcoming research for the strategical development of SFRC with innovative mechanical properties based on various limitations such as resource availability in the form of cost, material, time, and FS requirements for multiple construction projects.

This study is conducted to extract the effective ML approach to estimate the FS of SFRC precisely. The precise prediction of concrete characteristics would help one to obtain the durable structures’ economical, effective, and efficient design, ultimately reducing the time for selecting adequate materials and the resources, cost, and time. Furthermore, the SHAP analysis is conducted for depicting raw ingredients’ influence on SFRC FS, which has not been performed yet by the previous scholars and is the novelty of this work. The suggested prediction approaches would also assist scholars in the civil engineering field in developing new materials.

## 2. SHapley Additive ExPlanations (SHAP)

Moreover, in this work, the identification of global feature impacts and the relations of all the input features with FS of steel-fiber-reinforced concrete, based on game theory model (i.e., SHAP analysis) [41], is made for broadening the explainability of the suggested algorithm. In the procedure of SHAP analysis, each instance prediction is explicated by quantifying the features contribution by using SHapley values, attained by the employment of coalition of game theory. The average of all possible combinations for every feature value is taken to calculate the SHapley value. The SHapley values depict a direct relation with the feature influence. The global feature influence values are quantified by averaging all of the SHapley values of every database feature. Later, the descending order sorting, in terms of importance, for all values is done to draw a plot. A solitary point on the plot represents the SHapley value for each individual feature and instance. The X-axis shows the SHapley values and the y-axis portray feature importance. The position on the y-axis is directly related to feature influence on steel-fiber-reinforced concrete, where a color scale is used to indicate the feature importance. The plots of SHAP-feature dependence represents the interaction with/impact on steel-fiber-reinforced concrete, having colored the depiction for interactions. More elaborated information can be attained by using this method than partial dependence typical plots [40]. The feature importance (𝑗) for model f outcome; $\phi^{j}\left(f\right)\;$ is the assigned weight against feature contribution summation for output of model $f\left(x_{i}\right)\;$ for overall likely feature mixtures [42]. $\phi^{j}\left(f\right)\;$ is represented via Equation (1), as presented below:

(1) $$\phi^{j}\left(f\right)=\sum_{S\subseteq \left\{x^{1},\ldots \ldots ,x^{p}\right\}/\left\{x^{j}\right\}}\frac{\left|S\right|!\left(p-\left|S\right|-1\right)!}{p!}\left(f\left(S⊔\left\{x^{j}\right\}\right)-f\left(S\right)\right)$$

𝑆 = subset of features;$\;x_{j}$ = 𝑗 feature; and 𝑝 = the number of features in model. 

The SHAP technique determines the feature importance by quantifying the errors for prediction while distressing a specific feature value. The estimated error sensitivity is used for assigning weights to feature importance while perturbing its value. The trained ML model performance is explained by using SHAP. SHAP employs a method, i.e., input linear factors addition model demonstration, that is interpretable and is considered by the output of the model. For example, a model having input factors $x_{i}$, where the range of i is from 1 to k, k shows the number of input factor, and *h* ($x_{s}$) shows model explanation havng $x_{s}$ as an input, where Equation (2) is applied for the depiction of an original model f(x):

(2) $$f\left(x\right)=h\left(x_{s}\right)=\emptyset_{0}+\sum_{i=1}^{p}\emptyset_{i}x_{s}^{i}$$

where

p = number of input feature;

$\emptyset_{0}$= constant with no input.

**Figure 1:**
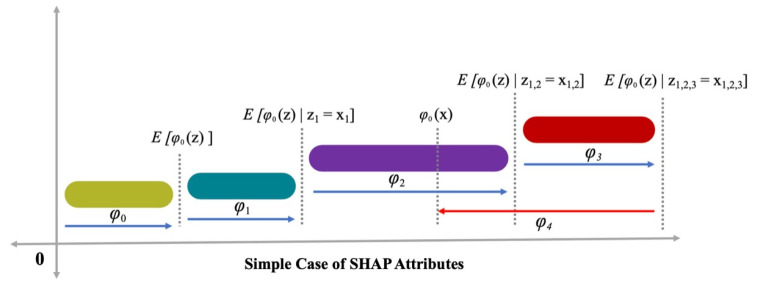
Attributes of SHAP analysis [44].

The mapping function, i.e., $x=m_{x}\left(x_{s}\right)$, has a relationship with input x and $x_{s}\;$ parameters. In the literature [43], Equation (2) is presented, where the prediction value, i.e., (*h* ()), was enhanced in terms of $\emptyset_{0},\;\emptyset_{1},\;\mathrm{and}\;\emptyset_{3}$, with an observed decrement of *h* () in terms of $\emptyset_{4}$, as presented in Figure 1. Three desired characteristics are included in Equation (2), in terms of consistency, local accuracy, and missingness. The reduction minus the attribution is ensured by consistency, that is, allocated to a relevant feature as a feature change of significant influence. In missingness, it is ensured to have no value for importance assigned to the features that are missing, such as $\emptyset_{i}=0$ is employed in terms of $x_{s}^{i}=0$. As far as local accuracy is concerned, it is ensured that the summation of features attribution will be taken as a function for the outcome, which requires a model to tie the outcome as a simplified input $x_{s}$ for f. $x=m_{x}x_{s}$ denotes the local precision achievement.

## 3. Dataset

**Figure 2:**
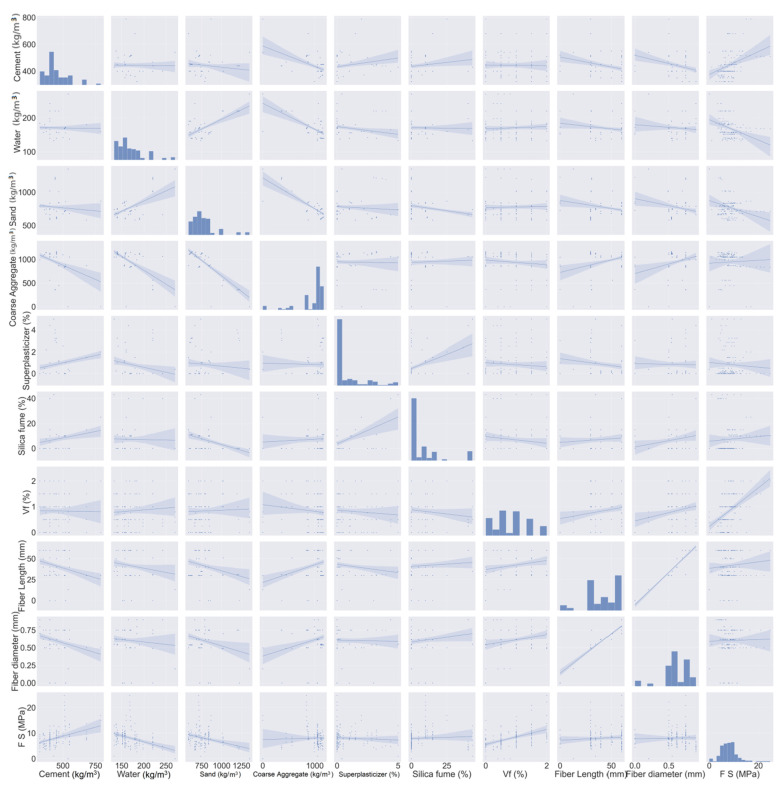
Input and output parameters.

**Figure 3:**
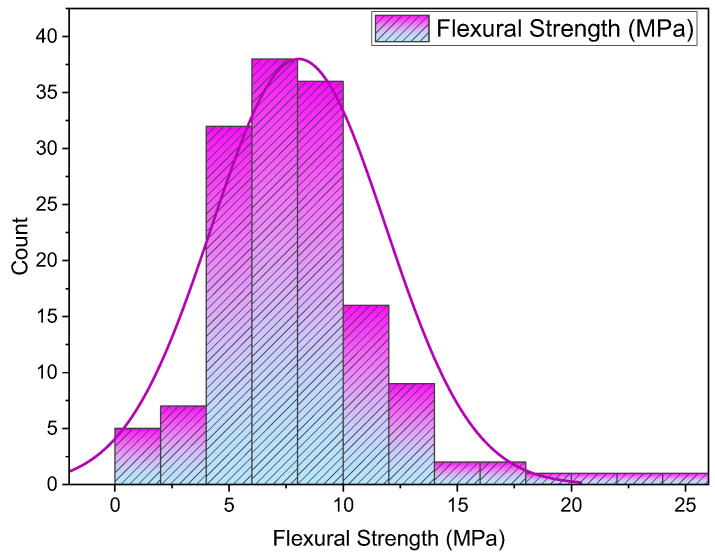
FS distribution.

The adopted dataset for estimating the FS of SFRC is depicted in Figure 2. The said dataset includes 151 mix designs with nine input parameters and is attained from the literature [34,45,46,47,48,49,50,51,52,53,54,55,56,57,58,59,60]. The factors cement (kg/m^3^), water (kg/m^3^), sand (kg/m^3^), coarse aggregate (kg/m^3^), superplasticizer (%), silica fume (%), Vf (%), fiber length (mm), and fiber diameter (mm). The variables for estimation in case of FS, which is considered as an output parameter in the current study, are based on input parameters. These variables are illustrated in Figure 2. Anaconda software’s Python and Spyder scripting are deployed for the estimation [61]. The histogram for FS being utilized in this work is presented in Figure 3. 

## 4. Results and Analysis

### 4.1. Decision Tree Adaptive Boosting

**Figure 4:**
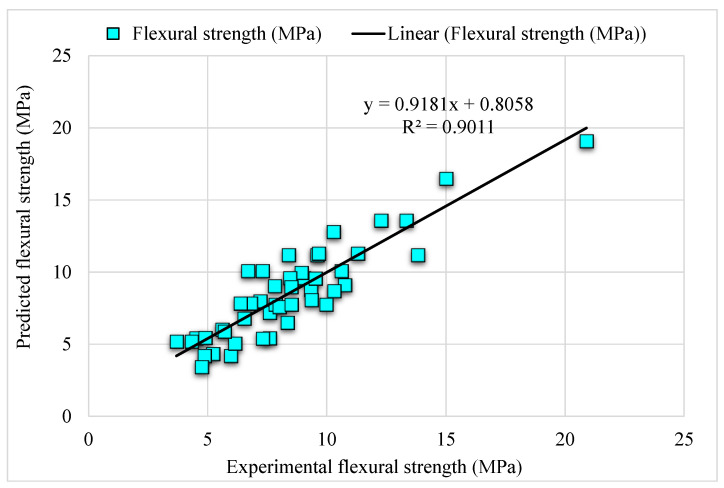
Experimental and AdaBoost predicted results.

**Figure 5:**
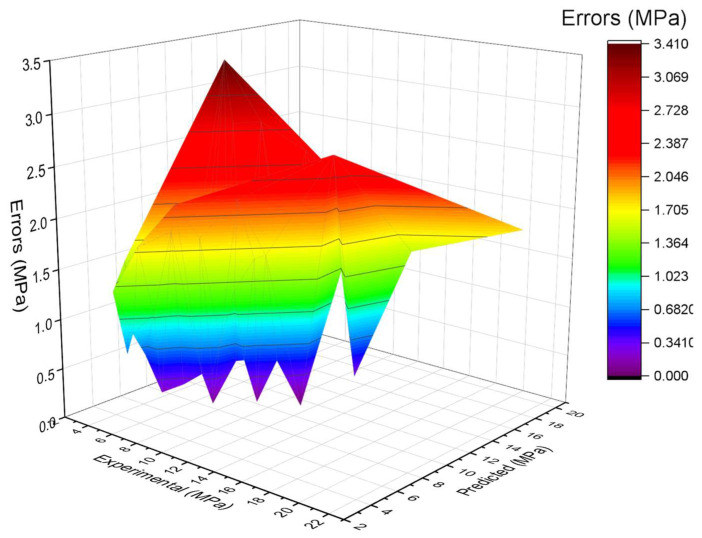
Estimated AdaBoost and experimental values, with errors.

The experimental and AdaBoost algorithm estimated values comparison for FS of SFRC is shown in Figure 4. Outcomes in the case of AdaBoost are reasonable, having less variation for SFRC FS. The 0.90 R^2^ value depicts the AdaBoost model’s suitability. Figure 5 shows the experimental and AdaBoost estimated error values distribution for SFRC FS. Figure 5 is plotted with an error difference between the experimental and predicted values on the Z-axis, while the X-axis shows the experimental values and Y-axis presents the predicted values. The error of experimental and estimated AdaBoost algorithm values for FS is 3.41 MPa, and 43% of values are less than 1 MPa, 39% of values are among 1 to 2 MPa, and 18% of values are more than 2 MPa. 

### 4.2. Decision Tree Bagging

**Figure 6:**
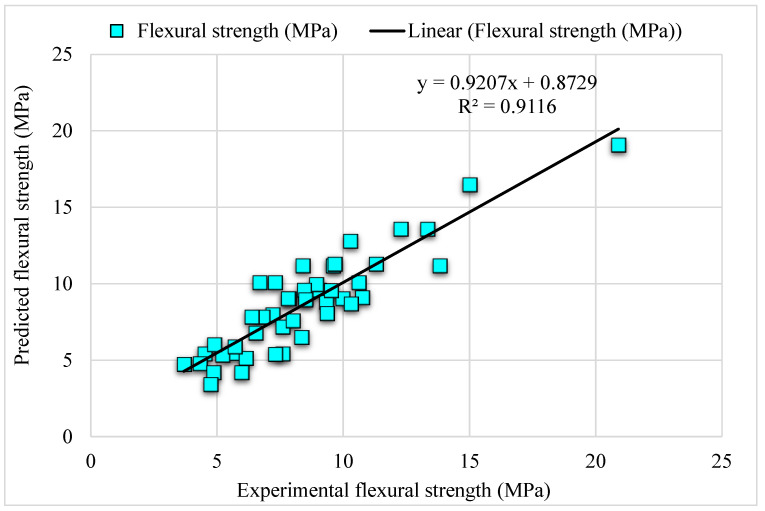
Experimental and bagging predicted results.

**Figure 7:**
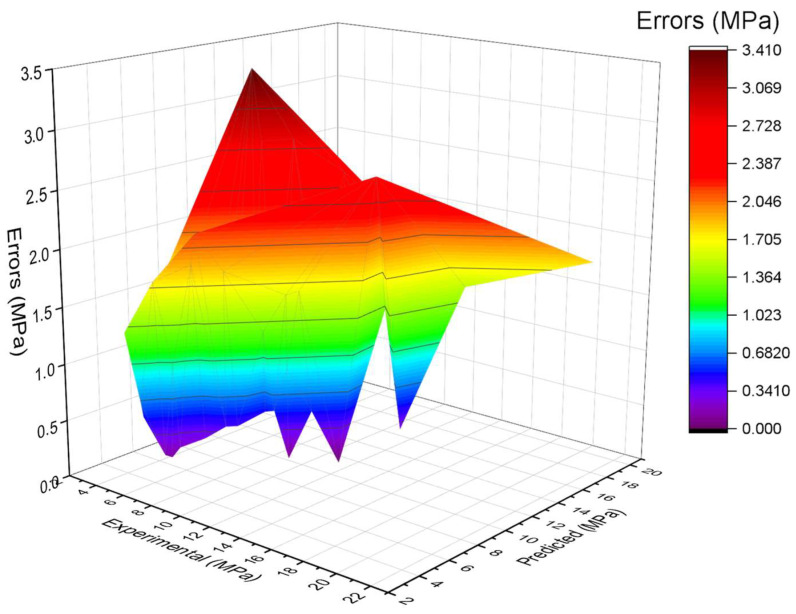
Estimated bagging and experimental values, with errors.

Figure 6 depicts the comparison of the bagging model experimental and predicted error values in the case of SFRC FS. The bagging R^2^ of 0.91 indicates highly precise outcomes than the AdaBoost model. Figure 7 illustrates the distribution of error in the case of experimental and bagging estimated values against SFRC FS. It may be noted that the error between experimental and estimated bagging algorithm values is 43% below 1 MPa; 43% is in the range from 1–2 MPa, followed by 13% values that are higher than 2 MPa. Higher R^2^ with a lesser error value for the bagging algorithm exhibits higher precision than AdaBoost. 

### 4.3. Gradient Boosting

**Figure 8:**
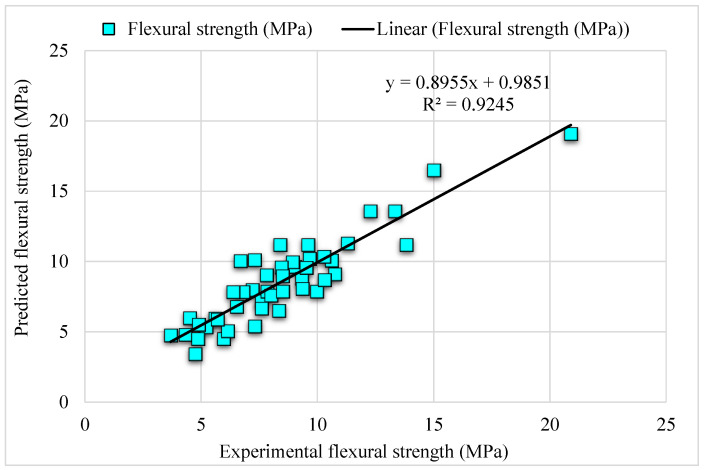
Experimental and gradient boosting predicted results.

**Figure 9:**
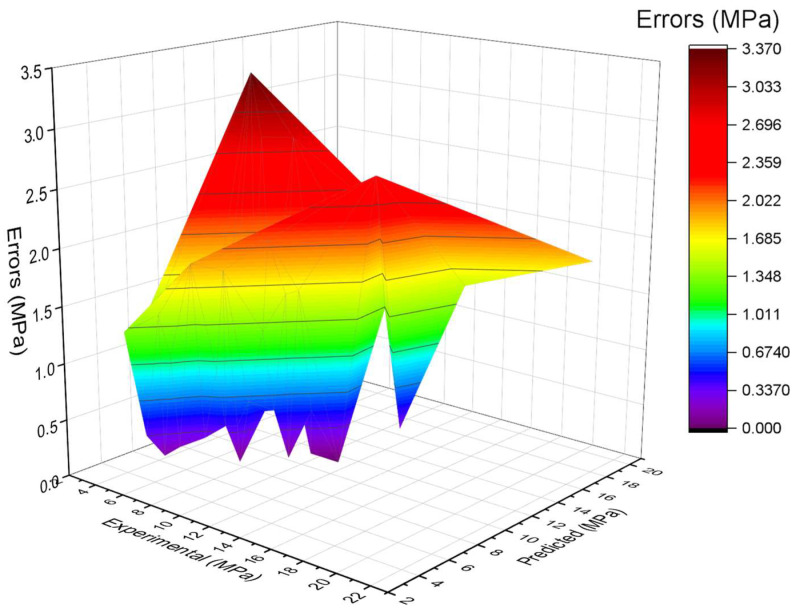
Estimated gradient boosting and experimental values, with errors.

Figure 8 represents gradient boosting predicted and experimental values for the output parameter of SFRC. The R^2^ of 0.92 indicates highly accurate gradient boosting outcomes as compared to the bagging model. Furthermore, it is a highly accurate model among all the other considered models. Figure 9 illustrates the experimental and bagging predicted errors distribution. It is noted that less than 1 MPa, there are 48% values; 50% from 1 to 2 MPa; and above 2 MPa, there are 2% values. In comparison with AdaBoost, the gradient boosting is more precise.

### 4.4. Extreme Gradient Boosting

**Figure 10:**
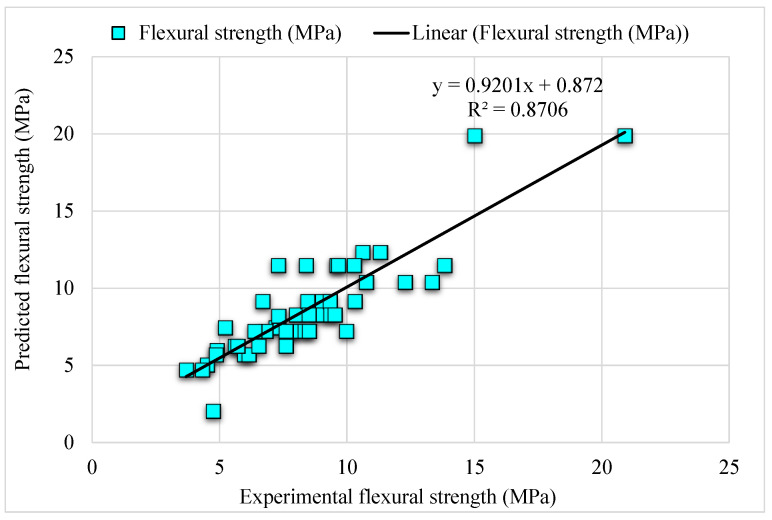
Experimental and estimated extreme gradient boosting outcomes.

**Figure 11:**
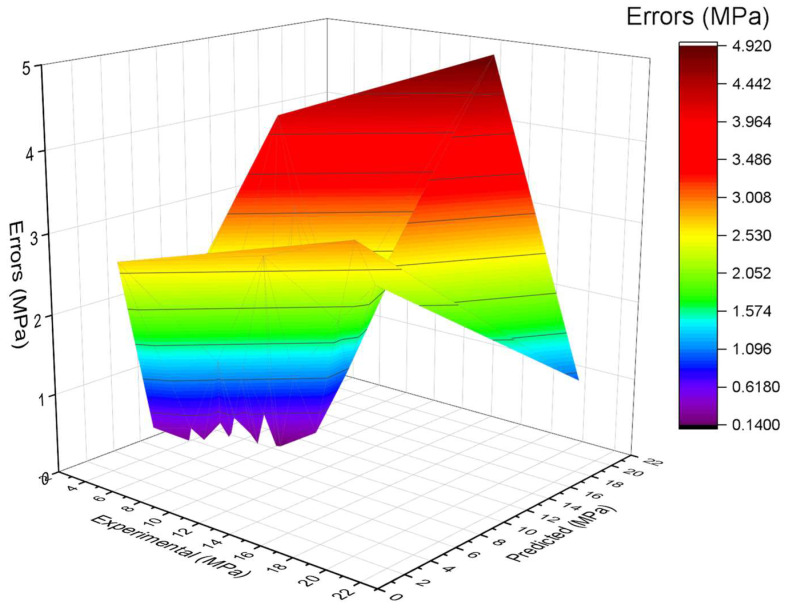
Estimated extreme gradient boosting and experimental values, with errors.

Figure 10 illustrates the experimental and estimated extreme gradient boosting values for SFRC considered output parameter. The R^2^ of 0.87 for extreme gradient boosting depicts lesser accuracy of outcomes than all other considered algorithms. The experimental and extreme gradient boosting predicted values error distribution for FS of SFRC is presented in Figure 11. Here, 50% of the values are below 1 MPa, 43% are 1 to 2 MPa, and the remaining 7% are above 2 MPa. Lower R^2^ and more error values portray unacceptable outcomes of the extreme gradient boosting algorithm than bagging, AdaBoost, and gradient boosting. However, the bagging model’s low error and higher R^2^ values are adequate and depict accurate prediction. Therefore, as per these findings, it can be said in the case of bagging that it may predict outcomes more accurately than all the considered models.

### 4.5. Comparison of All Models

**Figure 12:**
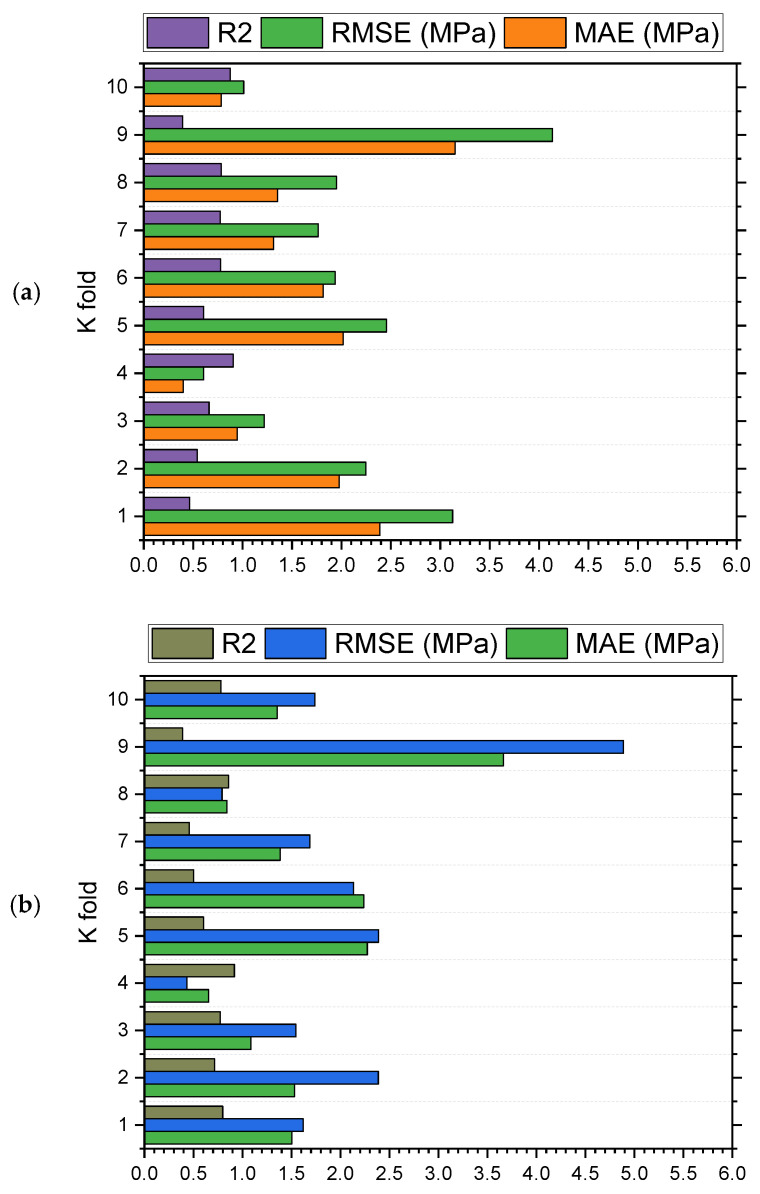

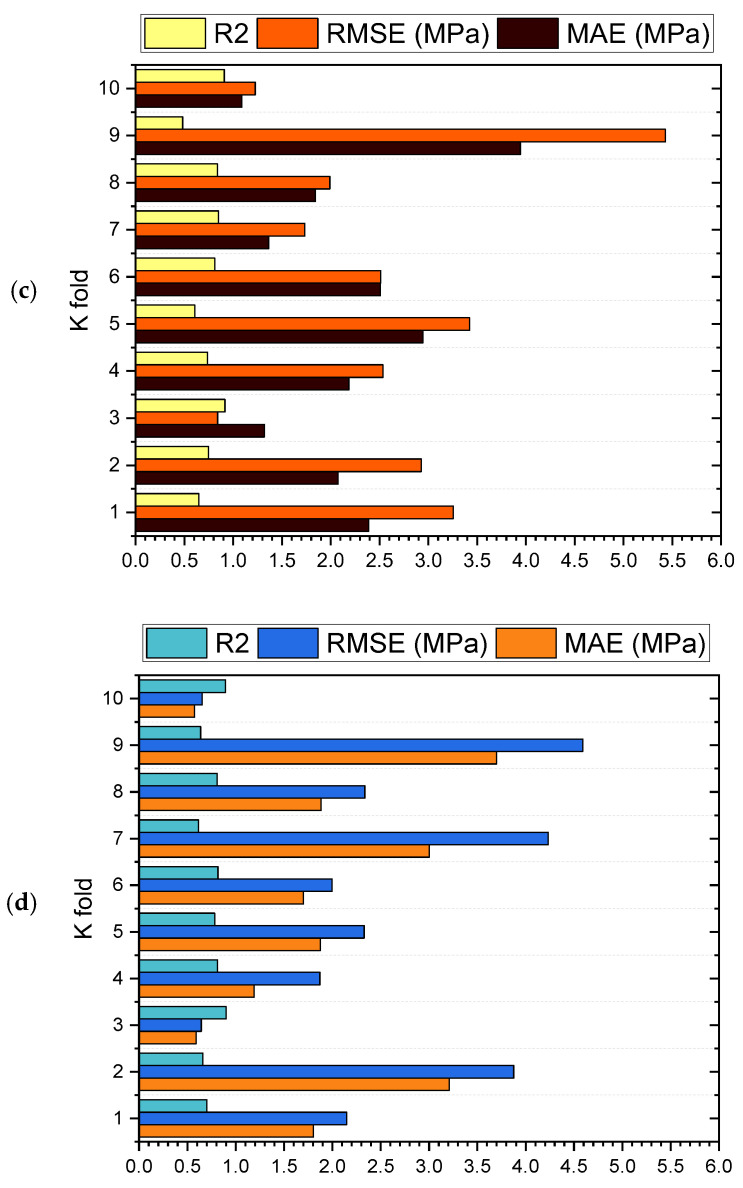
(**a**) AdaBoost; (**b**) bagging; (**c**) gradient boosting; and (**d**) extreme gradient boosting statistical representation.

The model’s validity is evaluated during the execution by applying the k-fold cross-validation technique. The performance of models is assessed with the help of statistical checks [36,37,38,39]. Generally, the splitting of data in a grouping of 10 for attaining the arbitrary scattering in k-fold cross-validation, and the ten-time repetition of this process, is done to obtain satisfactory outcomes. Table 1 illustrates the statistical checks for all the models. The R^2^ of 0.92, 0.87, 0.90, and 0.91 in the case of gradient, extreme gradient, adaptive boosting, and bagging models, as represented in Figure 12a–d. The MAE and RMSE are calculated by employing Equations (3) and (4), from the previous studies [36,37,38,39]. It is observed that the gradient boosting has a lower error and higher R^2^ values compared to all other considered models for SFRC flexural strength.

(3) $$\mathrm{MAE}=\frac{1}{n}\overset{n}{\underset{i=1}{\sum }}\left|x_{i}-x\right|$$

(4) $$\mathrm{RMSE}=\sqrt{\sum \frac{{\left(y_{pred}-y_{ref}\right)}^{2}}{n}}$$

where n = the total number of data, x, $y_{ref}$ = reference values of the data, and $x_{i}$, $y_{pred}$ = predicted model values.

**Figure 13:**
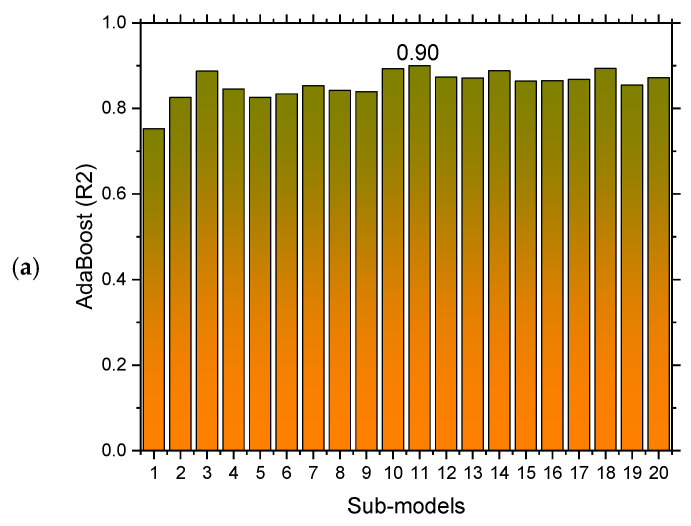

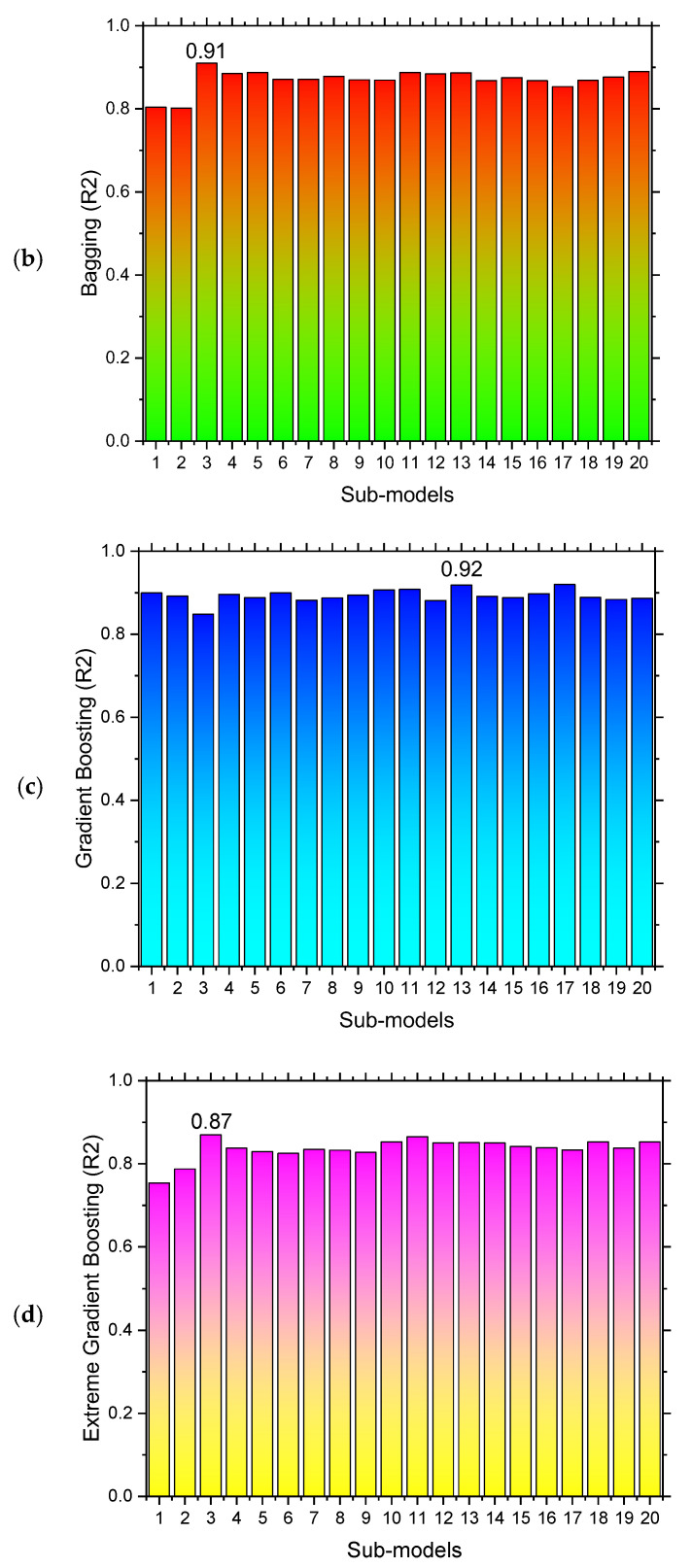
(**a**) AdaBoost; (**b**) bagging; (**c**) gradient boosting; and (**d**) extreme gradient boosting sub models’ outcomes.

SFRC FS is estimated by applying ensembled ML techniques in the current study, which is focused on providing reliable and efficient outcomes. The 0.92 R^2^ value for a gradient boosting result with the lowest MAE and RMSE have offered more precise estimations for the FS of SFRC. Out of 20 sub-models, an optimized model for SFRC FS prediction, as presented in Figure 13a–d, the ensembled ML gradient-boosting model has superior performance in terms of MAE (1.07) and RMSE (1.34). Therefore, it is depicted that, among all other models, the ensembled ML gradient-boosting model has provided the highest accuracy and lowest error.

### 4.6. Enhanced Explainability for Machine Leaning Algorithms

**Figure 14:**
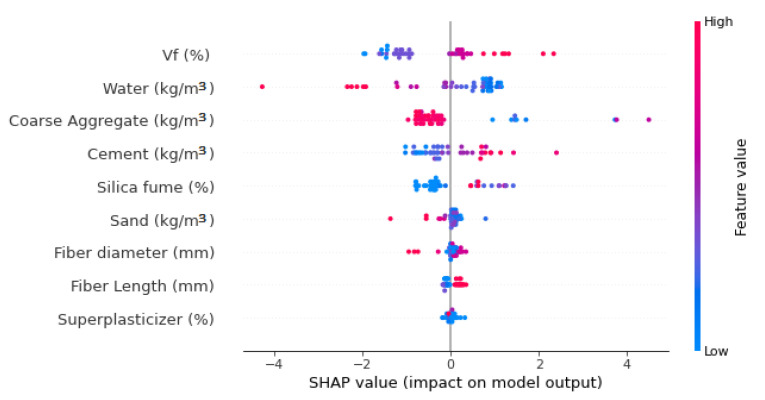
SHAP plot.

A detailed explanation of machine learning algorithms and features’ relations are presented in this work. At the start, by applying the SHAP tree explainer to the entire dataset, an enhanced illustration for influences of global features by incorporating SHAP explanations is also discussed. The SHAP method is applied [62]. The determination of the tree-based models’ internal structure is carried out with this method, summing up calculations set that is inter-connected with a leaf node of the tree model, resulting in low-order complexity [62]. The model interpretation is conducted for SFRC FS by using SHAP. The relation of different features with SFRC flexural strength is represented by SHAP values (Figure 14).

It is observed that the volumetric content of steel fiber has a maximum SHAP value for SFRC FS estimation as metallic fibers provide the effect of sewing, ultimately enhancing mechanical characteristics. Therefore, enhancing the steel fibers content would develop more SFRC FS, proving its positive influence. Figure 14 illustrates that the 2nd highest SHAP value is for water content. However, it has a negative influence, which means that increasing the amount of water causes decreased flexural properties of SFRC. In SFRC, the particle packing density theory is the basis of strength; hence, the requirement is to opt for limited water content in this case. Similarly, in the third, the coarse aggregates content also negatively influences SFRC FS. Then, the cement positively impacts the SFRC FS, which means that enhancement in cement content would increase the strength and vice versa. Further, the SFRC properties are significantly influenced by silica fume. Sand, however, shows both positive and negative influences, depending upon the content. Other features such as steel fibers diameter and length and super-plasticizer also have some minor but unique influences on SFRC FS. 

**Figure 15:**
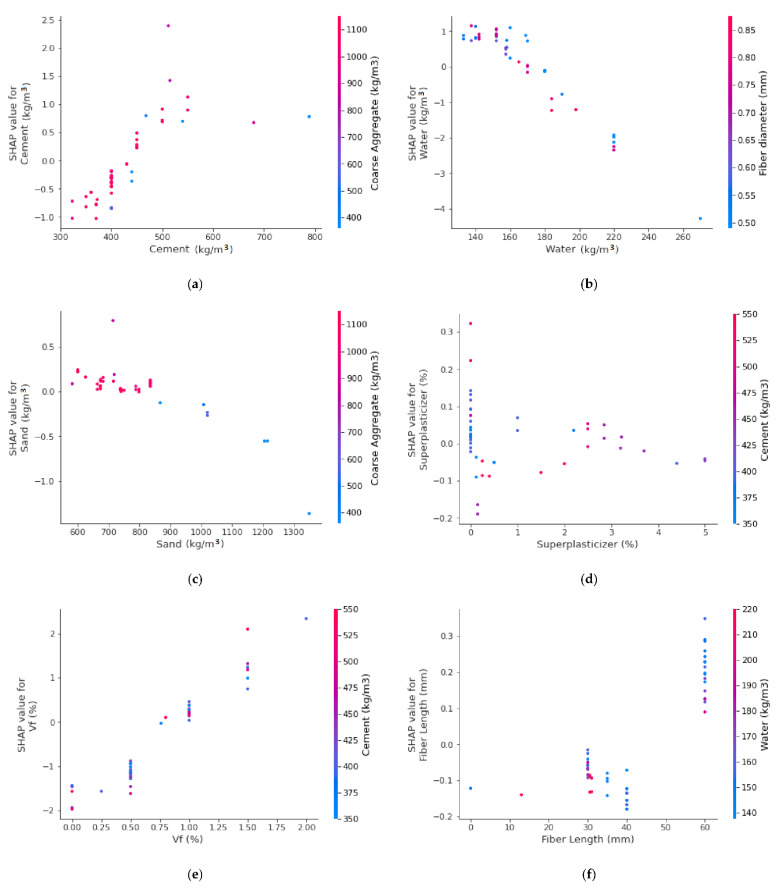
Interaction plot: (**a**) cement; (**b**) water; (**c**) sand; (**d**) superplasticizer; (**e**) Vf; and (**f**) fiber length.

The different features’ interaction with SFRC FS is depicted in Figure 15. The cement feature interaction is shown in Figure 15a. The amount of cement has major direct impact on SFRC FS. Figure 15b illustrates the negative impact of water for SFRC. Increasing the water content leads to decreasing SFRC FS, leading to a decreasing trend. The sand feature interaction is provided in Figure 15c. The sand content also represents a negative impact on SFRC. However, up to 800 kg/m^3^, it is not very effective on the FS of SFRC. Beyond this content of 1300 kg/m^3^, it causes a reduction in strength. This might be because more cement paste would be required to coat a larger surface area of sand particles in case of more sand content, ultimately leaving less cement to be accounted for in terms of strength development. Then, in a row, the super-plasticizer content feature depicts both negative and positive interactions, depending upon optimum content (Figure 15d). Up to 3% of the content contributes towards strength enhancement; however, beyond this content, it causes a reduction in strength. The steel fiber volumetric content feature positively influences up to 2% content (Figure 15e), showing its direct relationship with SFRC FS. Similarly, steel fiber length also positively influences and directly relates to SFRC FS, as is evident from Figure 15f. The greater length of steel fibers would enhance the SFRC flexural properties by providing a more effective bridging mechanism.

This prediction is based on the database utilized in the current study and focuses on strength prediction. However, the interaction between fiber length and diameter is found based on a limited data set in this study, and more accurate findings can be obtained by including more data points in the future. By expanding the number of data points, importing a slightly higher number of mixes, and taking into consideration a larger number of input factors (fiber length and diameter), a far more accurate model may be constructed for interaction. To improve the number of data points and outcomes in future studies, it is suggested that experimental work, field testing, and numerical analysis employing a range of approaches be implemented. The limitations of machine learning methods for estimating the strength properties of concrete have already been documented in a previous study [63].

## 5. Conclusions

Nowadays, the construction industry is focused on utilizing artificial intelligence (AI) approaches to estimate the mechanical properties of concrete. The main focus of this research is to evaluate the accuracy of AI approaches for predicting SFRC FS, in addition to exploring the raw components effect on SFRC flexural strength, which have not been studied yet and constitute a research gap. Nine estimation input parameters are considered, and their interaction is analyzed. Based on the conducted study, the following conclusions are drawn:

The gradient-boosting model’s higher R^2^ value of 0.92 depicts a highly precise estimation of flexural strength of SFRC out of the actual data, where the extreme gradient boosting, bagging, and AdaBoost have 0.87, 0.90, and 0.91 R^2^ values, respectively, in SFRC flexural strength prediction within an acceptable range. Twenty sub-models that range between 10–200 estimators are used to optimize the prediction of SFRC flexural strength. The most effective and accurate forecast for SFRC flexural strength emerged for gradient boosting rather than for the other considered algorithms.

The higher R^2^ and lower MAE and RMSE values for SFRC FS prediction from gradient boosting are evident from k-fold cross validation findings. Therefore, it can be claimed as the prediction model with the highest precision for flexural strength of SFRC.

Statistical checks such as MAE and RMSE are also applied to evaluate the models’ performance. Here again, the higher coefficient of determination and lower error values in gradient boosting for SFRC flexural strength prediction show their superiority.

Hence, it can be concluded that gradient boosting is the best technique for predicting SFRC flexural strength.

The volumetric content of steel fiber has the highest influence on SFRC flexural strength, followed by the contents of cement, water, and coarse aggregates, as revealed through SHAP observations. Contrary to this, super-plasticizer content has a minimal impact on SFRC flexural strength.

The feature interaction plot portrays that cement content is a major and positive influencing feature on SFRC flexural strength.

## Figures and Tables

**Table 1:** Extreme gradient boosting, bagging, and AdaBoost model statistical checks.

Statistical Checks	Approaches			
	Extreme Gradient Boosting	Decision Tree AdaBoost	Decision Tree Bagging	Gradient Boosting
R^2^	0.87	0.90	0.91	0.92
RMSE (MPa)	1.65	1.46	1.43	1.34
MAE (MPa)	1.26	1.21	1.18	1.07

## Data Availability

All data is available in the paper.
